# Psychometric validation of the English version of the digital wellbeing questionnaire for young adults

**DOI:** 10.1371/journal.pone.0346670

**Published:** 2026-04-15

**Authors:** Magdalena Liberacka-Dwojak, Germano Vera Cruz, Monika Wiłkość-Dębczyńska, Merve Aktaş Terzioğlu, Andrée-Anne Légaré, Laura Orsolini, Todd Farchione, Tania Lecomte, Sandy Ingram, Riaz Khan, Yasser Khazaal

**Affiliations:** 1 Department of Psychology, Kazimierz Wielki University, Bydgoszcz, Poland; 2 Department of Psychology, UR 7273 CRP-CPO, University of Picardie Jules Verne, Amiens, France; 3 Department of Child and Adolescent Psychiatry, Pamukkale University Faculty of Medicine, Denizli, Türkiye; 4 Department of Community Health Sciences, Faculty of Medicine and Health Sciences, Université de Sherbrooke – Longueuil, Québec, Canada; 5 Unit of Clinical Psychiatry, Department of Neurosciences/DIMSC, Polytechnic University of Marche, Italy; 6 Department of Psychology, University of Boston, Boston, United States of America; 7 Department of Psychology, University of Montreal, Montreal, Canada; 8 School of Engineering and Architecture, HES-SO University of Applied Sciences of Western Switzerland, Fribourg, Switzerland; 9 Frontier Medical College affiliated to Bahria University Islamabad, Islamabad, Pakistan; 10 Addiction Medicine, Department of Psychiatry, Lausanne University Hospital and Lausanne University, Lausanne, Switzerland; 11 Research Centre, University Institute of Mental Health at Montreal and Department of Psychiatry and Addiction Montreal University, Montreal, Canada; South China Normal University, CHINA

## Abstract

**Background:**

Digital technologies increasingly impact daily life. Digital wellbeing is emerging as a multidimensional construct encompassing emotional regulation, autonomy, social connection, and respectful online interactions.

**Objective:**

This study aimed to validate the English version of the 13-item Digital Wellbeing Questionnaire (DWBQ) in young adults. Methods: Data were collected via quota sampling on Prolific, an online crowdsourcing platform. The final sample comprised 1,853 young adults (US n = 933, UK n = 920), including 892 women, 871 men, and 90 non-binary participants., aged 18–25. Confirmatory factor analysis (CFA), measurement invariance testing (across gender and country), reliability analysis, and convergent validity assessments (with the Digital Flourishing Scale and the Digital Stress Scale) were conducted.

**Results:**

CFA confirmed the four-factor structure (emotional resilience, agency, social connection, and communion), with good model fit (CFI = .969, TLI = .959, RMSEA = .058, SRMR = .038). The DWBQ showed strong internal reliability (α ≥ .80) and measurement invariance across both gender and country. Convergent validity was supported by positive correlations with digital flourishing and negative associations with digital stress. Sociodemographic factors and smartphone use patterns were significantly associated with DWBQ subscales, with gender and relationship status particularly influencing agency and emotional resilience

**Conclusions:**

The English version of the 13-item DWBQ is a valid and reliable measure of digital wellbeing among young adults in the US and UK. It offers a concise tool for research, educational, and clinical use.

## 1. Introduction

Smartphone usage has seen a dramatic rise worldwide, increasing from 1.57 billion users in 2014–7 billion in 2023 [1]. Recent study shows that young adults now spend nearly five hours per day on smartphones, surpassing traditional TV viewing [2]. It is driven by short-form video platforms such as TikTok and Instagram Reels, which are associated with compulsive engagement and regretful use [3,4]. Problematic smartphone use has been reported across multiple countries and in numerous studies. A study of a representative sample of the American adult population recently reported, using stringent criteria, a rate of problematic smartphone use of 0.75% to 1.2% [5–7]. While smartphones offer numerous conveniences, such as access to social media, dating apps, gaming, and other practical resources, their pervasive presence has raised concerns about their impact on individual wellbeing. Constant connectivity and the availability of online interactions can affect attention, emotional regulation, life satisfaction [8] and interpersonal relationships, particularly among younger users [9].

Recent research suggests that excessive smartphone use may negatively affect psychological health, as well as academic and social functioning [10–12]. In particular, problematic engagement with short-form video platforms has been linked to increased stress, diminished sense of control, impaired social interaction, and difficulties in academic achievements [4,6,10,12]. In addition, Problematic Social media use, often accessed through smartphones, has been linked to similar challenges [13]. Such technological immersion may also reduce focus during offline social interactions. Adolescents and young adults (ages 15–18) appear particularly vulnerable to problematic smartphone use [14,15], a vulnerability that challenges in self-regulation and impulse control may partly explain, fear of missing out, distress, depression, among others [8,16,17]. However, young adulthood represents a distinct developmental stage characterized by increasing autonomy, identity consolidation, and ongoing maturation of self-regulatory capacities. Recent studies indicate that individuals aged 18–25 show heightened vulnerability to dysregulated smartphone and social media use, partly due to ongoing development of impulse control and emotional regulation [13]. In addition, recent artificial intelligence related developments and the wide dissemination of Large Language Models (LLM) tools within the population came with new opportunities and risks [18–20]. Adolescence is furthermore a developmental period characterized by greater autonomy, exposure to new social and emotional challenges, and loneliness-related risks [21,22], which may compromise the ability to regulate smartphone use effectively.

In light of these findings, the concept of digital wellbeing has been proposed as distinct from mental wellbeing [23–25]. Mental wellbeing refers to a broad state of psychological functioning that includes emotional balance, life satisfaction, coping capacity, and the ability to function productively and meaningfully in everyday life) [26–28]. By contrast, digital wellbeing refers more specifically to how individuals experience and manage their functioning within technology-mediated environments [29]. It captures the ability to regulate digital habits, maintain agency over screen use, and engage in positive and respectful online interactions [30,31]. Accordingly, digital wellbeing could be seen as a multidimensional construct encompassing emotional regulation, agency, and social connection, aligning with recent models of technology use and wellbeing. These dimensions are reflected in the Digital Wellbeing Questionnaire [32]. It reflects the extent to which digital engagement supports or undermines balance, autonomy, emotional stability, and the quality of online social interactions. Rather than being defined only by the absence of problematic use, digital wellbeing is increasingly conceptualized as a multidimensional construct that includes both adaptive and maladaptive aspects of digital engagement [29,31].

Overall, research suggests that individuals engage with smartphones in both adaptive and maladaptive ways. While some usage patterns enhance social support, access to information, and entertainment [33], others contribute to stress, distraction, digital stress, and a diminished sense of control [34,35]. This duality highlights the importance of further exploring the multidimensional nature of the digital wellbeing construct.

Recent conceptualizations of digital wellbeing emphasize the importance of emotional regulation in response to digital interactions, referring to an individual’s ability to maintain psychological stability and effectively manage emotional responses triggered by online experiences [31]. In addition, individuals need to have a sense of control over their screen, digital habits, and the boundaries to self-regulate their smartphone use. This aligns with self-determination theory that emphasizes autonomy as a key component of wellbeing [36,37]. Beyond self-regulation, digital wellbeing also depends on the quality of social connections facilitated through online interactions. Recent studies show that constructive and prosocial online engagement predicts higher digital satisfaction and wellbeing [30,38]. Taken together, these conceptual developments suggest that digital wellbeing should be assessed not only through problematic or excessive use, but also through perceived emotional resilience, agency, and the quality of online relational experiences. This makes the availability of concise and psychometrically robust multidimensional instruments especially important.

Several instruments have been proposed to assess digital wellbeing, including the Perceived Digital Well‑Being Scale (PDWS) [39] and the Digital Flourishing Scale (DFS) [33]. However, these measures emphasize somewhat different aspects of digital experience. The PDWS focuses primarily on perceived balance and satisfaction with digital use, whereas the DFS centers more strongly on positive perceptions of mediated social interactions and flourishing in digital contexts [33,40]. These measures provide however limited coverage of emotional regulation and relational norms in relation to digital experiences.

One of the few instruments specifically designed to assess digital wellbeing that includes these aspects is the 13-item Digital Wellbeing Questionnaire (DWBQ; Priyanka, 2023). The Digital Wellbeing Questionnaire (DWBQ) places greater emphasis on emotional regulation and relational norms in the assessment of digital wellbeing compared to other instruments such as the Perceived Digital Well-Being Scale (PDWS). The DWBQ was developed using a mixed-methods approach and validated to capture four distinct domains: emotional resilience (which directly assesses emotional regulation), agency, social connection, and communion. These domains specifically address how individuals manage emotions and maintain healthy relational norms in digital contexts, which are central to digital wellbeing but underrepresented in other scales [32]. The DWBQ’s multidimensional structure allows for a more comprehensive evaluation of digital wellbeing, particularly in the domains of emotional regulation and relational norms, which have been identified as pivotal in digital competency [32,30,41].

The present study aimed to: (a) assess the factor structure and the psychometrics properties of the Digital Wellbeing Questionnaire (DWBQ; [32]) among a relatively large sample of USA and UK young adult participants (including structure validity and its invariance concerning country and gender; (b) test the scale internal reliability and convergent validity in relation to other two scales designed to measure close constructs, namely the Digital Flourishing Scale (DFS; [33,40]) and the Multidimensional Digital Stress Scale (DSS; [34]); (c) explore the relationships between the DWBQ dimensions and the participants’ sociodemographic characteristics and smartphone use behavior.

## 2. Methods

### 2.1. Recruitment and sampling

This study used a cross-sectional online survey design. Participants were anonymously recruited through Prolific, an online crowdsourcing platform that provides access to a large, diverse, and pre-screened participant pool. Prolific adheres to established ethical research standards, including informed consent and data quality assurance [42,43]. Participants received monetary compensation in line with Prolific’s standard remuneration rates for online research studies. Prolific applies prescreening filters, unique participant identifiers, and IP-based controls to prevent multiple participation [44].

Inclusion criteria were: being between 18 and 25 years old, using a smartphone and social media daily, and being fluent in English. The data collection started on 29.03.2025 and finished on 11.04.2025. To ensure a gender-sensitive approach, a quota sampling strategy was employed, aiming to recruit 2,160 participants with a distribution of 42% men, 42% women, and 16% non-binary individuals.

The target sample size was set to exceed recommended thresholds for confirmatory factor analysis (CFA), namely a minimum of 200 participants and at least 10 participants per estimated parameter (Kline, 2016). The final sample consisted of 1,853 young adults (US n = 933, UK n = 920), representing 85% of the intended recruitment target and substantially exceeding psychometric requirements. No cases were excluded due to missing data.

Participants were excluded if they (a) failed attention checks, (b) provided incomplete demographic or questionnaire data, or (c) did not meet the age criterion. After exclusions, the final analytic sample comprised N = 1,853 participants. There were no missing data.

### 2.2. Data collection material

The data were collected via an online questionnaire, a task outsourced to Prolific, as explained above. All questionnaires used in this study are publicly available for academic purposes. The questionnaire included:

1. *Socio-demographic questions,* including: age, gender (male, female, non-binary), relationship status (not in relationship, in relationship but not married, in relationship and married), level of education (measured in terms of years of schooling), and socio-economic status (SES; low, intermediate, high). SES was assessed using a single self-report item in which participants categorized their perceived socio-economic position as low, intermediate, or high. These three categories were not constructed from composite indicators but reflected participants’ subjective evaluation of their economic situation, consistent with common practice in online survey research.
2. *Smartphone and social media behavior*, all indicators of smartphone use were based on self-reported estimates. Participants were asked to report:
  a. the average daily time spent on a smartphone for activities that are not essential during the last three months, and
  b. the first and second most frequently used applications.
3. The expression “activities that are not essential” referred to unnecessary use such as non-instrumental social networking, entertainment, browsing, or gaming,.
4. *The Digital Wellbeing Questionnaire* (DWBQ; [32] a 13-item scale to measure users’ subjective digital wellbeing across four dimension: *emotional resilience* (ability to regulate emotions online, 5 items), *agency* (sense of control over screen use, 2 items), *social connection* (feeling supported and connected, 3 items), *communion* (experiencing kindness and respect online, 3 items), rated on a 7-point Likert scale. Higher scores indicate greater digital wellbeing. The scale internal consistency dimensions were: *α* = .85,.87,.85,.82, respectively. The DWBQ does not provide validated clinical or normative cutoff scores; therefore, the scale is intended to be interpreted as a continuous measure of digital well-being rather than as a categorical classification. This approach is consistent with the original development and validation of the instrument.
5. *The Digital Flourishing Scale* (DFS; [33,40]), a 25-item self-report measure to assess digital flourishing among five dimensions: *connectedness* (combination of social connection and social support), *self-control, civil participation*, *positive social comparison*, and *authentic self-disclosure,* rated on a 7-point Likert scale, from 1 = strongly disagree to 7 = strongly agree. Higher scores indicate higher flourishing. The scale internal consistency dimensions were: *α* = .91,.82,.86,.92,.87, respectively.
6. *The Multidimensional Digital Stress Scale* (DSS; [34]), a 23-item self-report measure to assess digital stress among five dimensions: *connection overload*, *availability stress, approval anxiety*, *fear of missing out*, *online vigilance* rated on a 7-point Likert scale, from 1 = strongly disagree to 7 = strongly agree) was applied. Higher scores indicate higher digital stress. The scale internal consistency dimensions were: *α* = .89,.87,.91,.86, and 85, respectively.

### 2.3. Ethics

The study was carried out following the Declaration of Helsinki. Bioethics Committee of the Nicolaus Copernicus University at Collegium Medicum in Bydgoszcz, Poland (approval no. KB 100/2025) approved the study protocol. Prior to participation, individuals received detailed information about the purpose, procedures, and voluntary nature of the study. Participants provided digital informed consent by clicking to proceed to the next section of the online survey, which was clearly indicated as a confirmation of consent. The ethics committee approved this method of consent. The survey was fully anonymous and did not involve the collection of identifying information. No minors were included in the study.

### 2.4. Participants

Table 1 presents descriptive statistics for US and UK participants. The total sample consisted of 1853 participants with a mean age of 22.4 years (SD = 2.10). Both samples were similar in age, education, and gender distribution. Most identified as heterosexual, were single or in an informal relationship, and rated their socio-economic status as intermediate. Among the U.S. participants, the most frequently reported first app of the day was TikTok, used by 23.3% of the total sample. This was followed by Instagram, selected 14.6%, and YouTube, reported by 7.8%. The most frequently reported second app among U.S. users was Instagram, selected by 15.8%, followed by TikTok with 14.3% and YouTube, used by 9.4%. Similarly, in the U.K. sample, TikTok was also the most commonly reported first app, indicated by 24.0% of participants, followed by Instagram with 18.9% of users and YouTube, which was selected by 8.3% of participants. The most frequently used second app in the U.K. was Instagram, reported by 13.3% participants, followed by TikTok, 10.5% users, and YouTube, mentioned by 5.7% participants.

**Table 1:** Descriptive statistics of the participants socio-demographic characteristics and smartphone use behavior.

Variable	USA sample (*n* = 933)		UK sample (*n* = 920)	
	Mean	Standard deviation	Mean	Standard deviation
Age	22.40	2.08	22.30	2.11
Education level (years of education)	15.40	2.96	16.10	2.64
	Frequency	Percentage	Frequency	Percentage
Gender				
Female	444	47.60	448	48.70
Male	436	46.70	435	47.30
Non-binary	53	5.70	37	4.00
Sexual orientation				
Heterosexual	656	70.40	676	73.50
Homosexual	64	6.90	65	7.10
Bisexual	168	17.90	146	15.90
Other	45	4.80	33	3.60
Relationship status				
Not in a relationship	395	42.30	448	48.70
In relationship, but not married	406	43.50	438	47.60
In relationship, married	132	14.10	33	3.60
Perceived socio-economic status				
Low	248	26.60	266	28.90
Intermediate	631	67.60	612	66.50
High	54	5.80	42	4.60
	Mean	Standard deviation	Mean	Standard deviation
Smartphone time use	6.95	3.87	6.13	3.22

### 2.5. Statistical analyses

All analyses were conducted in SPSS v29 and AMOS v29. Descriptive analysis included mean (M), standard deviation (SD), skewness, and kurtosis. Our psychometric testing followed established best-practice protocols for scale validation [45]. First, data suitability was examined using the Kaiser-Meyer-Olkin (KMO) test, and sphericity using Bartlett’s test was conducted.

We then conducted confirmatory factor analysis (CFA) with maximum likelihood estimation, reporting multiple model fit indices (χ²/df, CFI, TLI, RMSEA, SRMR) in line with recommended cutoffs, values of CFI and TLI ≥ .95 were considered indicative of good fit (≥.90 acceptable), RMSEA ≤ .06 and SRMR ≤ .08 indicated good fit, and a χ²/df ratio < 5 was taken as acceptable [46]. Reliability was assessed using Cronbach’s α, with values exceeding.70 consider acceptable, and corrected item–total correlations [47]. Convergent validity was tested through correlations with the Digital Flourishing Scale (DFS) and the Digital Stress Scale (DSS). Finally, multi-group CFA was used to assess measurement invariance across gender and country. This sequence aligns with the six-step protocol for psychometric validation outlined by Dima (2018), ensuring transparency and reproducibility of results. Configural, metric, and scalar models were estimated sequentially. Invariance was determined based on ΔCFI ≤ .01 [48].

## 3. Results

### 3.1. Descriptive statistics of the DWBQ items

Table 2 summarizes item-level descriptive statistics for the DWBQ, organized by its four subscales. Most items showed moderate means and acceptable skewness and kurtosis, indicating a relatively normal distribution of responses across every subscale.

**Table 2:** Descriptive statistics of the DWBQ (13 items grouped into 4 factors).

Factors/ Items	Scale/Range	Mean	SD	Skewness	Kurtosis
**Factor 1: State of emotional resilience**					
ER3. I feel emotionally stable when I am exposed to the digital world.	1-7	4.90	1.510	−.516	−.414
ER6. I can manage any emotional challenges caused by my screen use.	1-7	5.00	1.505	−.261	−.133
ER7. I cope well with any mood swings that are aroused by my screen use.	1-7	5.16	1.392	−.689	.143
ER8. I cope well with the emotional harm caused by screen use.	1-7	5.10	1.437	−.621	−.122
ER9. I cope well with any digital harm that comes my way. (e.g., cyberbullying).	1-7	5.22	1.557	−.722	−.202
**Factor 2: State of agency**					
A1. I set boundaries over my screen use.	1-7	4.07	1.791	−.059	−1.024
A2. I limit my screen time.	1-7	3.51	1.839	.282	−1.002
**Factor 3: State of social connection**					
C1. I feel good when others connect with me in the digital world.	1-7	5.11	1.341	−.602	.202
C2. I feel good when someone online reaches out to me during difficult times.	1-7	5.39	1.369	−.879	−.611
C3. I feel good when someone interacts with me in the digital world.	1-7	5.21	1.302	−.678	.419
**Factor 4: State of communion**					
C5. I feel good when online fellow users exhibit respectable conduct.	1-7	5.63	1.206	−.740	.342
C6. I feel good when online fellow users consider the appropriateness of the content before sharing.	1-7	5.66	1.238	−.712	.247
C7. I feel good when the people I meet online are kind to me.	1-7	5.83	1.173	−.991	.820

SD = standard deviation; DWBQ = Digital Wellbeing Questionnaire.

### 4.1. Confirmatory factorial analysis (CFA) on the DWBQ data

The adequacy of the sample for factor analysis was confirmed by the KMO measure (.818). Bartlett’s test of sphericity was highly significant, χ²(78) = 1200.15, p < .001, confirming that the correlations between items were sufficiently large to proceed with factor analysis.

**Fig 1:**
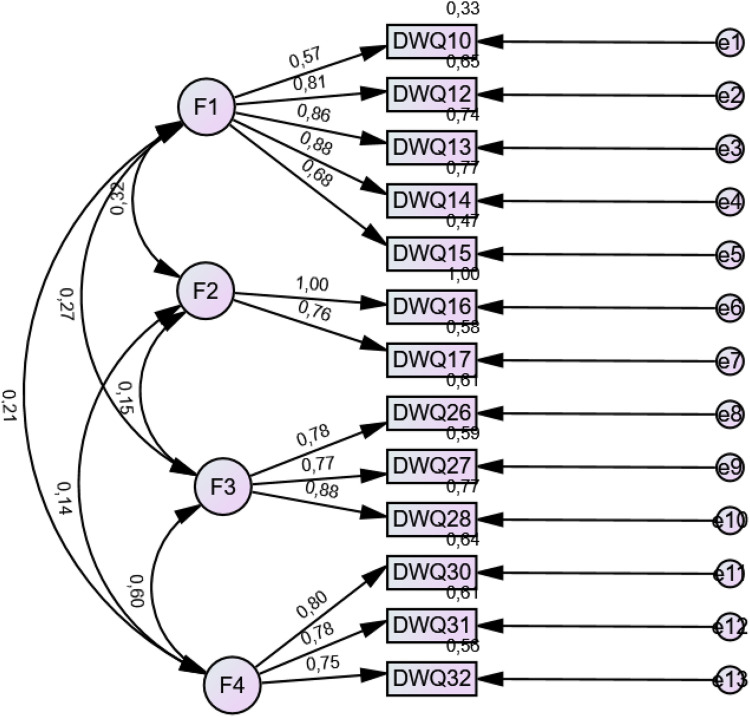
The CFA path diagram with the standardized estimates.

A confirmatory factor analysis (CFA) was conducted on the 13-item version of the DWBQ (see Fig 1). The model included 13 observed variables and four correlated latent factors (F1–F4). The model demonstrated a good fit to the data: χ²(60) = 436.37, p < .001; RMSEA = .058, 90% CI [.053,.063], PCLOSE = .004; CFI = .969; TLI = .959 and SRMR = .038. These indices indicate an acceptable to excellent model fit.

All standardized factor loadings ranged from.57 to.91 and were statistically significant (p < .001), indicating that each item was a strong indicator of its respective latent factor. The observed variables also showed significant variance (p < .001), supporting sufficient variability across items. In the initial estimation, a Heywood case was detected for the residual variance of DWQ16. To obtain an admissible solution, this variance was constrained to a small positive value (.001), a standard procedure in CFA when only two indicators represent factors. After applying this constraint, the model converged normally without inadmissible estimates and the global fit indices remained virtually unchanged. To enhance transparency, a path diagram of the final four-factor CFA model is presented in Fig 1 and standardized loadings are reported in Table 3.To strengthen the empirical justification for the hypothesized four-factor structure, alternative measurement models were tested and compared with the proposed model. Specifically, three competing models were examined: (1) an unidimensional model in which all 13 items loaded on a single latent factor, (2) a second-order hierarchical model with four first-order factors loading on a general digital wellbeing factor. The unidimensional model demonstrated very poor fit to the data: χ²(65) = 6481.48, p < .001; RMSEA = .231, 90% CI [.226,.236], PCLOSE < .001; CFI = .463; TLI = .356; SRMR = .194. The second-order model demonstrated acceptable fit: χ²(61) = 563.77, p < .001; χ²/df = 9.24; RMSEA = .067, 90% CI [.062,.072], PCLOSE < .001; CFI = .958; TLI = .946; SRMR = .064.

**Table 3:** Standardized factor loadings for the 13-item DWBQ four-factor CFA model (N = 1853).

Item	Factor	Standardized loading (β)	Unstandardized B	SE	CR	p	R²
DWQ10	F1	.573	1.000	–	–	–	.33
DWQ12	F1	.808	1.148	.046	25.21	<.001	.65
DWQ13	F1	.860	1.273	.049	26.06	<.001	.74
DWQ14	F1	.875	1.338	.051	26.27	<.001	.77
DWQ15	F1	.685	1.134	.050	22.78	<.001	.47
DWQ16	F2	1.000	1.000	–	–	–	.99*
DWQ17	F2	.762	0.783	.015	50.61	<.001	.58
DWQ26	F3	.784	1.000	–	–	–	.61
DWQ27	F3	.768	0.999	.030	33.29	<.001	.59
DWQ28	F3	.876	1.085	.030	36.36	<.001	.77
DWQ30	F4	.801	1.000	–	–	–	.64
DWQ31	F4	.784	1.005	.032	31.54	<.001	.61
DWQ32	F4	.749	0.909	.030	30.53	<.001	.56

Note. β = standardized factor loading; B = unstandardized regression weight; SE = standard error; CR = critical ratio; R² = proportion of variance explained. Dashes indicate fixed reference parameters. *Residual variance for DWQ16 was constrained to a small positive value (.001) to resolve an initial Heywood case. Parameters without SE, CR, and p-values were fixed to 1.00 for model identification and serve as reference indicators for each latent factor.

### 4.2. Country (USA vs. UK) invariance tests

A multi-group analysis was performed to indicate whether the DWBQ factor structure is invariant across countries.

A multi-group confirmatory factor analysis was conducted to evaluate measurement invariance of the DWBQ across countries. The configural model demonstrated good fit, CFI = .967, TLI = .956, RMSEA = .047 [90% CI.039–.056], SRMR = .079, supporting equivalence of the factor structure between the USA and UK samples. The metric model, in which factor loadings were constrained to equality across groups, also showed acceptable fit, CFI = .967, TLI = .959, RMSEA = .047 [90% CI.037–.055]. The chi-square difference between the configural and metric models was non-significant, Δχ²(9) = 1.74, p = .294, indicating that metric invariance was supported. Subsequently, scalar invariance was tested by constraining item intercepts to be equal across countries. The scalar model retained adequate fit, CFI = .954, RMSEA = .046. Changes in fit indices relative to the metric model were within recommended thresholds (ΔCFI = −.007; ΔRMSEA = .002), supporting scalar invariance. These findings indicate that the DWBQ demonstrates full measurement invariance across the USA and UK samples, permitting meaningful comparisons of latent means between countries.

### 4.3. Gender (female vs. male) invariance tests

A multi-group confirmatory factor analysis was conducted to evaluate measurement invariance of the DWBQ across gender. The configural model demonstrated good fit, CFI = .971, TLI = .962, RMSEA = .040 [90% CI.036–.044], SRMR = .045, supporting the equivalence of the factor structure between women and men. The metric model, in which factor loadings were constrained to be equal across groups, also showed acceptable fit, CFI = .971, TLI = .964, RMSEA = .039 [90% CI.035–.042], SRMR = .045. The chi-square difference between the configural and metric models was non-significant, Δχ²(9) = 14.46, p = .107, indicating that metric invariance was supported. Subsequently, scalar invariance was tested by additionally constraining item intercepts to equality across groups. The scalar model demonstrated good fit, χ²(142) = 664.51, CFI = .954, RMSEA = .046 [90% CI.042–.049]. Changes in model fit relative to the metric model were within recommended thresholds (ΔCFI = −.010; ΔRMSEA = .004), supporting scalar invariance. These results indicate that item intercepts are equivalent across women and men and that meaningful comparisons of latent means between genders are warranted.

### 4.4. Internal reliability

Table 4 summarizes the main results of the internal reliability tests conducted for each DWBQ subscale. Cronbach’s alpha indicated that DWBQ had good international reliability ≥ 0.80.

**Table 4:** Reliability statistics and item-total correlation.

Factors/ Items	Sub-scale Corrected Item-Total Correlation	Sub-scale Variance if Item Deleted	Sub-scale Cronbach’s Alpha if Item Deleted
**Factor 1: State of emotional resilience**			
ER3. I feel emotionally stable when I am exposed to the digital world.	.429	.314	.813
ER6. I can manage any emotional challenges caused by my screen use.	.597	.592	.800
ER7. I cope well with any mood swings that are aroused by my screen use.	.595	.648	.800
ER8. I cope well with the emotional harm caused by screen use.	.601	.667	.799
ER9. I cope well with any digital harm that comes my way. (e.g., cyberbullying).	.450	.434	.811
**Factor 2: State of agency**			
A1. I set boundaries over my screen use.	.419	.316	.815
A2. I limit my screen time.	.586	.588	.826
**Factor 3: State of social connection**			
C1. I feel good when others connect with me in the digital world.	.516	.629	.806
C2. I feel good when someone online reaches out to me during difficult times.	.466	.509	.810
C3. I feel good when someone interacts with me in the digital world.	.489	.616	.808
**Factor 4: State of communion**			
C5. I feel good when online fellow users exhibit respectable conduct.	.413	.499	.814
C6. I feel good when online fellow users consider the appropriateness of the content before sharing.	.426	.508	.813
C7. I feel good when the people I meet online are kind to me.	.413	.499	.814

SD = standard deviation; **α =** Cronbach’s alpha; DWBQ = Digital Wellbeing Questionnaire

### 5.1. Correlations between the DWBQ subscales

All dimensions of the DWBQ were significantly interrelated. Emotional resilience showed positive associations with agency, social connection, and communion. Similarly, agency correlated positively with social connection and communion. The strongest relationship was observed between social connection and communion (see Table 5).

**Table 5:** Correlation matrix of the DWBQ dimensions.

Factors	State of emotional resilience	State of agency	State of social connection	State of communion
State of emotional resilience	1	.259**	.252**	.162**
State of agency	.259**	1	.133**	.114**
State of social connection	.252**	.133**	1	.519**
State of communion	.162**	.114**	.519**	1

**Correlation is significant at the 0.01 level (2-tailed); *Correlation is significant at the 0.05 level (2-tailed); DWBQ = Digital Wellbeing Questionnaire

### 5.2. Convergent validity

The results indicate that all dimensions of digital wellbeing were positively associated with digital flourishing factors. In contrast, digital stress factors such as approval anxiety, fear of missing out, and online vigilance were negatively related to emotional resilience and agency. Total scores for digital flourishing were related to higher digital wellbeing, while the total digital stress score showed negative associations with most wellbeing components (see Table 6).

**Table 6:** Correlations between the three DWBQ factors, the DFS factors, and DS factors.

Scales	DWBQ				
	State of emotional resilience	State of agency	State of social connection	State of communion	DWBQ total score
DFS Factors					
Connectedness	.204**	.191**	.575**	.378**	.490**
Self-Control	200**	.197**	.559**	.360**	.480**
Civil Participation	.205**	.193**	.387**	.336**	.412**
Positive Social Comparison	.274**	.260**	.320**	.174**	.398**
Authentic Self-Disclosure	.526**	.284**	.305**	.289**	.529**
DFS Total score	.472**	.342**	.524**	.396**	.651**
DSS Factors					
Connection overload	−.225**	.087	−.002	.023	−.030
Availability stress	−.117**	.083	.150	.023	.061
Approval anxiety	−.295**	−.128**	.174*	.115	−.074
Fear of Missing Out	−.335**	−.178**	.051	.008*	−.191**
Online vigilance	−.237**	−.184**	.177*	.082	−.091**
DSS Total score	−.325**	−.090**	.149	.068	−.089**

**Correlation is significant at the 0.01 level (2-tailed). *Correlation is significant at the 0.05 level (2-tailed); DWBQ = Digital Wellbeing Questionnaire; DFS = Digital Flourishing Scale; DSS = Multidimensional Digital Stress Scale.

### 5.3. Relationships between DWBQ factors and the participants’ sociodemographic and smartphone use variables

For the ANOVA analysis, the app responses were grouped into four categories: the three most frequently used apps (TikTok, Instagram, and YouTube) and a general other category comprising all remaining responses. Statistical analyses revealed that gender, sexual orientation, and relationship status were significantly associated with several dimensions of digital wellbeing, with the highest effect size observed for the effect of relationship status on the DWBQ agency dimension. Smartphone screen time showed significant negative correlations with agency and positive correlations with social connection and communion. Education level was positively correlated with emotional resilience and agency. Among group comparisons, significant differences across digital wellbeing factors were also found for perceived socio-economic status and the most frequently used applications (see Table 7).

**Table 7:** Association between the three DWBQ factors, the participants’ sociodemographic characteristics, and smartphone use behavior.

Variable	State of emotional resilience	State of agency	State of social connection	State of communion	DWBQ total score
**Correlations (Pearson’s r)**					
Age	−.019	.030	−.017	.013	.024
Smartphone time use	−.044	−.108**	.129**	.234**	.181**
Education level (years of education)	.064**	.062**	.004	.017	.042
**Group comparisons (ANOVA)**					
Gender	F(2.1852) = 19.992. η² = .021**	F(2.1852) = 3.105. η² = .003*	F(2.1852) = 1.216. η² = .001*	F(2.1850) = 20.334. η² = .022**	F(2.1852) = 3.565. η² = .004*
Sexual orientation	F(3.1849) = 4.986. η² = .008**	F(3.1849) = 4.162. η² = .007**	F(3.1849) = 1.480. η² = .002*	F(3.1849) = 4.470. η² = .007**	F(3.1849) = 1.195. η² = .002*
Relationship status	F(2.1851) = 5.197. η² = .006**	F(2.1851) = 39.917. η² = .041**	F(2.1851) = 5.974. η² = .006**	F(2.1851) = 5.556. η² = .007**	F(2.1851) = 31.146. η² = .033**
Perceived socio-economic status	F(2.1852) = 3.859. η² = .004*	F(2.1852) = 19.774. η² = .021**	F(2.1852) = 3.607. η² = .004*	F(2.1852) =.520. η² = .001*	F(2.1852) = 14.114. η² = .015**
First app most used	F(3.1849) = 1.070. η² = .002*	F(3.1849) = 15.111. η² = .024**	F(3.1849) = 1.465. η² = .002*	F(3.1849) = 1.146. η² = .002*	F(3.1849) = 5.382. η² = .009**
Second app most used	F(3.1849) = 1.198. η² = .000	F(3.1849) = 3.171. η² = .005*	F(3.1849) =.524. η² = .001	F(3.1849) =.290. η² = .001	F(3.1849) =.909. η² = .001

**Correlation is significant at the 0.01 level (2-tailed). *Correlation is significant at the 0.05 level (2-tailed); DWBQ = Digital Wellbeing Questionnaire; DFS = Digital Flourishing Scale; DSS = Multidimensional Digital Stress Scale.

## 4. Discussion

The present study aimed to validate the English version of the Digital Wellbeing Questionnaire (DWBQ) among young adults in the United States and the United Kingdom, evaluating its factorial structure, reliability, measurement invariance, and convergent validity. Findings support the DWBQ as a psychometrically sound and theoretically grounded tool for assessing multidimensional wellbeing.

The CFA supported the hypothesized four-factor structure, comprising emotional resilience, agency, social connection, and communion. This pattern is theoretically meaningful because it suggests that digital wellbeing includes both intrapersonal and interpersonal dimensions of functioning in digital environments. In other words, wellbeing in the digital sphere is not limited to self-control or the absence of distress, but also includes perceived emotional stability, a sense of agency over technology use, and the quality of online relational experiences. This interpretation is consistent with prior work emphasizing the complexity of digital engagement and its self-regulatory and socio-affective components [32,49]. The structure captures how technology can foster not only negative effects but also positive psychological outcomes, such as support or kindness [50]. Importantly, the emotional and relational components captured by the DWBQ highlight that sensitivity and empathy are relevant for online experiences [31]. The DWBQ’s conceptual foundation aligns with self-determination theory, which highlights autonomy and competence as central factors affecting wellbeing [37,51], as well as with broader wellbeing models such as PERMA, which emphasize positive emotions, engagement, relationships, meaning, and accomplishment [52]. This confirms the four-factor structure comprising emotional resilience, agency, social connection, and communion.

Further, the internal consistency of the DWBQ subscales supports the reliability of the constructs in culturally distinct Western countries. Measurement invariance analyses demonstrated acceptable to good model fit across countries (USA vs. UK) and genders (female vs. male), with non-significant differences in chi-square values, indicating configural and metric invariance. This suggests that young users from different Western countries interpret digital wellbeing similarly [53], highlighting the tool’s generalizability and potential use in cross-cultural research. The DWBQ’s consistent results across both genders underscore that the scale assesses the construct with similar capability across the male and female genders. Although non-binary participants were included in the overall sample, their numbers were insufficient for invariance testing.

Subscale intercorrelations among subscales support the socio-affective components of digital interaction [54,55], especially considering the strong correlation between social connection and communion (r = .519). This may suggest that individuals who feel a strong sense of belonging and support in digital spaces are also more likely to engage in respectful and prosocial interactions online. This association highlights the intertwined nature of relational digital experiences.

Convergent validity was supported by positive correlations between DWBQ subscales and the DFS, and negative associations with the DSS. These findings suggest that digital wellbeing is associated not only with lower digital stress but also with higher levels of fulfilling, socially meaningful online experiences. Wellbeing in the digital context is related to self-perceived connectedness and significant engagement [37,51] and is negatively associated with stressors such as fear of missing out or approval anxiety [34]. Notably, emotional resilience and agency were most sensitive to digital stressors, suggesting they may serve as psychological buffers of online engagement [36]. However, the correlations between the DWBQ dimensions, the DSS, and background characteristics were generally small in magnitude. This suggests that digital wellbeing reflects primarily subjective and contextual processes rather than simple exposure or demographic indicators.

Digital wellbeing was differentiated by several sociodemographic and behavioral variables. Gender differences emerged across all subscales, with small-to-moderate effect sizes (η² = .021–.022), particularly in emotional resilience and communion, indicating that females may be more vulnerable to socio-emotional dynamics online. Similarly, relationship status was most strongly associated with agency (η² = .041), potentially reflecting the role of interpersonal contexts in shaping control over digital habits. Smartphone use time was positively associated with social connection and communion but negatively associated with agency, highlighting the ambivalent role of screen engagement [56]. Longer screen time may facilitate online bonding but also change one’s sense of self-regulation [57]. Furthermore, higher education levels were positively correlated with emotional resilience and agency, suggesting that cognitive resources may buffer against negative digital impacts. These patterns reflect the broader context of how digital technologies are related to individual differences, including vulnerabilities and protective factors. For example, individuals lacking agency may be more prone to compulsive use, while those with strong emotional regulation capacities may be less reactive to online comparison [58,59].

The validation of the DWBQ offers a reliable tool for assessing digital wellbeing across diverse populations. It also supports the conceptualization of digital wellbeing as both an individual and relational construct. The DWBQ is well-suited for use in clinical, educational, and research contexts and is easy to administer in survey-based research. These findings reinforce the view that technologies are not inherently harmful or addictive; rather, wellbeing depends on how individuals engage with them, balancing engagement, emotion regulation, and interpersonal connection.

Compared to Priyanka’s initial validation study, this study expands the scope of DWBQ by validating it across two countries (the United States and the United Kingdom), using a substantially larger sample focused specifically on individuals aged 18–25. Moreover, measurement invariance tests confirmed the scale’s stability across gender and the use of related instruments such as DFS and DSS to measure construct validity.

Despite its strengths, the study has several limitations. The cross-sectional design limits interpretations. Longitudinal research is necessary to assess the temporal dynamics of digital wellbeing. Moreover, validation of the DWBQ in other age groups and non-Western cultures may be beneficial, as cultural norms may influence perception of digital life. Future research should also explore clinical applications of the DWBQ, including its relationships with depression, anxiety, or compulsive smartphone or social media use. Moreover, the use of online, non-probability quota sampling may limit the generalizability of the findings due to possible self-selection bias [60], attracting individuals with specific interests or characteristics that do not reflect the broader population. At the same time, the large sample size (N = 1853) and balanced representation across gender and country strengthen the psychometric validation. In addition, the study did not assess test–retest reliability, leaving the temporal stability of the scale unexamined, and relied solely on self-report data without behavioral measures of technology use. In addition, the use of online quota sampling may introduce self-selection bias, as individuals recruited via digital platforms may differ systematically from the general population in digital literacy and engagement patterns [60]. This may limit generalizability beyond digital active young adults. Several other methodological limitations should be acknowledged. First, one Heywood case was observed, suggesting mild model misspecification and warranting cautious interpretation of the corresponding parameter estimates. Second, although overall model fit was acceptable, the RMSEA approached the upper bound of recommended cutoffs, indicating that model fit, while adequate, was not optimal. Third, although configural, metric, and scalar invariance were supported across country and gender, invariance could not be tested for non-binary participants due to limited sample size, restricting conclusions regarding gender-diverse groups. Finally, the validation was conducted in a non-clinical population, so further research is warranted to evaluate the instrument in clinical contexts.

Despite these limitations, the study offers important implications. For practice, the DWBQ can serve as a screening tool to identify individuals at risk of impaired digital wellbeing. For research, our findings support extending validation to diverse populations and longitudinal designs. For education, the DWBQ can inform digital literacy programs that emphasize balanced technology use among students. Finally, for administration and policy, the questionnaire can guide institutional monitoring and initiatives aimed at promoting healthier digital habits. More broadly, these findings support the idea that digital wellbeing should not be reduced to exposure indicators such as screen time alone. The relatively modest associations with background variables and the differentiated pattern across subscales suggest that digital wellbeing is shaped not only by how much technology is used, but also by how it is experienced, regulated, and embedded in social interaction. This has important implications for both research and practice, as it argues against overly simplistic interpretations of digital life as either uniformly harmful or uniformly beneficial.

It is important to note that the present dataset overlaps with data used in a recently published study [61]. However, the two publications address distinct instruments, constructs, and research questions. The first study focused on the Perceived Digital Wellbeing Scale, whereas the present manuscript provides psychometric validation of the DWBQ. This complementary publication strategy enables the examination of two distinct instruments while laying a robust scientific foundation for future research initiatives. value. Further studies may assess how the different measures converge and diverge across populations, contexts, and outcomes, and examine their relative sensitivity, predictive validity, and utility for research, prevention, and clinical practice.

## Conclusions

The present study provides strong psychometric evidence for the validity and reliability of the English 13-item Digital Wellbeing Questionnaire (DWBQ) among young adults in the United States and the United Kingdom. While the findings support the reliability of the scale, the cultural generalizability should be made with caution given the use of non-probability quota sampling and restriction to US and UK samples. Future research should extend validation to other age groups, cultural contexts, clinical populations, and explore longitudinal application. However, the DWBQ has potential as a practical tool for educators and clinicians to monitor and promote balanced digital engagement in young adults.
